# HIF‐2α regulates non‐canonical glutamine metabolism *via* activation of PI3K/mTORC2 pathway in human pancreatic ductal adenocarcinoma

**DOI:** 10.1111/jcmm.13202

**Published:** 2017-05-24

**Authors:** Wenzhu Li, Changhao Chen, Xiaohui Zhao, Huilin Ye, Yue Zhao, Zhiqiang Fu, Wenwei Pan, Shangyou Zheng, Lusheng Wei, Tianwen Nong, Zhihua Li, Rufu Chen

**Affiliations:** ^1^ Department of Oncology Sun Yat‐sen Memorial Hospital Guangzhou China; ^2^ Department of Urology Sun Yat‐Sen Memorial Hospital Guangzhou China; ^3^ Department of Hepatopancreatobiliary Surgery Sun Yat‐sen Memorial Hospital Guangzhou China; ^4^ Department of Tumor Intervention The First Affiliated Hospital of Sun Yat‐Sen University Guangzhou China; ^5^ Department of Gynaecology and Obstetrics The First Affiliated Hospital of Sun Yat‐sen University Guangzhou China

**Keywords:** HIF‐2α, pancreatic ductal adenocarcinoma, non‐canonical glutamine metabolism, pathway

## Abstract

Hypoxia‐inducible factor‐2α (HIF‐2α) plays an important role in increasing cancer progression and distant metastasis in a variety of tumour types. We aimed to investigate its biological function and clinical significance in human pancreatic ductal adenocarcinoma (PDAC). A total of 283 paired PDAC tissues and adjacent normal tissues were collected from patients who underwent surgery or biopsy at Sun Yat‐sen Memorial Hospital between February 2004 and October 2016. In this study, we noted that HIF‐2α expression was significantly up‐regulated in PDAC, positively associated with disease stage, lymph‐node metastasis and patient survival, and identified as an independent prognostic factor of PDAC patients. We demonstrated that HIF‐2α silencing could reduce proliferation, migration and invasion of PDAC cells *in vitro*. The similar effect on growth was demonstrated *in vivo*. Furthermore, we noted that knock‐down of HIF‐2α significantly decreased the expression of glutamate oxaloacetate transaminase 1 (GOT1). Importantly, we confirmed that the PI3K/mTORC2 pathway promoted GOT1 expression by targeting HIF‐2α. Our study validated HIF‐2α was an important factor in PDAC progression and poor prognosis and may promote non‐canonical glutamine metabolism *via* activation of PI3K/mTORC2 pathway. Targeting HIF‐2α represents a novel prognostic biomarker and therapeutic target for patients with PDAC.

## Introduction

Pancreatic cancer is a typical hypoxia digestive solid malignancy. It is so lethal that the 5‐year survival rate is only around 5% 1, 2. Aberrant metabolism is considered as one of the hallmarks of cancer 3. A remarkable phenomenon named Warburg effects is exploited in the clinic for diagnostic purposes, which is exhibited in various tumours 4, 5. Previous research showed that human PDAC utilizes a special metabolism pathway, a non‐canonical pathway of glutamine, in which glutamine‐derived aspartate is converted into oxaloacetate by aspartate transaminase (GOT1), and this oxaloacetate is converted into malate and then pyruvate, increasing the NADPH/NADP^+^ ratio which can potentially maintain the cellular redox state 6, 7. Reprogramming of energy metabolism is the hallmark progression in the research of malignancy neoplasm 3. It can balance energy metabolism and biosynthesis to maintain the high malignancy behaviours 8.

The phosphatidylinositol 3‐kinase (PI3K)/AKT/mTOR pathway plays a significantly role in pancreas in accelerating the formation of PDAC 9, 10, activating in premalignant pancreatic lesions and pancreatic cancer tissues 10, 11, 12, and regulating migration, metabolism, autophagy, survival and growth 13. PI3K/AKT/mTOR pathway regulates the uptake and utilization of multiple nutrients, including glucose, glutamine, nucleotides and lipids, in a manner best suited for supporting the enhanced growth and proliferation of cancer cells 14. The mammalian target of rapamycin (mTOR) is a catalytic subunit and forms two complexes: mTORC1 and mTORC2, mTOR binds Raptor forms mTORC1, whereas mTOR binds Rictor forms mTORC2 15, 16. In some human tumours, like neuroblastoma and renal cell carcinoma, HIF‐1α expression was depending on mTORC1, in contrast, mTORC2 regulates the transcription levels of HIF‐2α 15, 17, 18, 19.

Hypoxia is another important feature of solid tumours. It has been convinced that a series of molecular biology change induced by hypoxia are the important reasons of higher malignant, like proliferation, invasion, migration and chemoradiotherapy resistant, relating to hypoxia‐inducible factor (HIF) signalling pathway 20, 21. HIFs are composed of two heterodimeric proteins and contain three subunits, HIF‐1α, HIF‐2α and HIF‐3α; among them, HIF‐1α and HIF‐2α are best studied so far 22. HIF‐1α protein and HIF‐2α protein were detected in most types of human tumours, including pancreatic, bladder, colon, breast, glial, hepatocellular, prostate and renal tumours 23. Different to HIF‐1α, HIF‐2α is active under mild or physiological hypoxia (<5% O_2_), and active even after 48–72 hrs 24. Some important cellular activities are regulated by HIF‐2α, such as proliferation, invasion, differentiation and metabolism 25. Despite that the effect of hypoxia in glycolysis has been verified 26, 27, the effect of HIF‐2α on non‐canonical pathway of glutamine is still unknown in PDAC.

In this study, we investigated HIF‐2α expression in PDAC tissues and analysed its correlation with the clinicopathological characteristics and prognosis of PDAC patients. We also studied the function and mechanism of HIF‐2α in PDAC cells. Our findings strongly indicate that HIF‐2α participates in PDAC carcinogenesis and could regulated non‐canonical glutamine metabolism by activation of PI3K/mTORC2 pathway. We believe that HIF‐2α is a potential diagnostic and prognostic marker and a promising therapeutic target.

## Materials and methods

### Patients and clinical samples

The 283 paired PDAC samples were obtained from patients who underwent resection or biopsy at Sun Yat‐sen Memorial Hospital between February 2004 and October 2016. No patient received anticancer treatment before surgery. Table 1 presents the detailed data of the clinicopathological characteristics. All samples were evaluated and histologically diagnosed by expert pathologists. All samples were collected with informed consent according to the Sun Yat‐Sen University internal review and ethics boards.

**Table 1:** Correlation between HIF‐2α expression and clinicopathological characteristics of PDAC patients

Characteristics	Patient frequency (%)	HIF‐2α level		
		Low	High	*P* value†
Total cases	283	135	148	
Gender				
Male	167	80	87	0.935
Female	116	55	61	
Age				
<60	126	61	65	0.593
≥60	157	71	86	
Differentiation				
Well	77	50	27	0.001**
Moderate	123	62	61	
Poor	83	23	60	
TNM stage(AJCC)				
I	24	14	10	0.006**
II	172	93	79	
III	23	7	16	
IV	64	21	43	
T stage				
T1	4	4	0	0.024*
T2	33	19	14	
T3	211	102	109	
T4	35	11	24	
Lymph‐node metastasis				
Negative	113	66	47	0.003**
Positive	170	69	101	
Perineural invasion				
Negative	133	82	51	0.001**
Positive	150	63	87	

^†^Chi‐square test, *P < 0.05, **P < 0.01.

### Cell culture

The pancreatic cell lines of Capan‐2 and Panc‐1 were provided by the American Type Culture Collection (ATCC, Manassas, VA, USA). Cells were cultured in DMEM (Gibco, Grand Island, NY, USA) with 10% foetal bovine serum (FBS) and 1% penicillin/streptomycin in a 37°C incubator in a humidified atmosphere of 5% CO_2_. For the hypoxia treatments, cells were incubated in an anaerobic chamber (Invivo2400, Ruskinn Technology, Sanford, Maine, USA). Cells were treated with LY294002 (20 μM, Selleckchem), rapamycin (1 μM, Selleckchem), GDC‐0068 (1 μM, Selleckchem) or PP242 (1 μM, Selleckchem).

### RNA isolation and quantitative RT‐PCR (qRT‐PCR)

Total RNA was isolated from tissues or pancreatic cell lines using a standard Trizol protocol (Invitrogen, Carlsbad, CA, USA). Total RNA was converted to cDNA by reverse‐transcription using oligodT primers and SuperScript II reverse transcriptase (Invitrogen, San Diego, CA, USA). Real‐time PCR was performed using SYBR Green reaction mix (Qiagen, Germantown, MD) and analysed on a Roche Light‐Cycler system (Roche, Basel, Switzerland). β‐Actin was used as the normalization control. The qRT‐PCR data were analysed and showed as the fold change (2^−ΔΔCT^). The qRT‐PCR reaction for each sample was repeated in triplicate. The primer sequences were showed in Table S1.

### Cell transfection and viral infection

For transient knock‐down experiments, the following small interfering RNAs (siRNAs) were used: HIF‐2α siRNA (si‐HIF‐2α) and synthetic sequence‐scrambled siRNA (si‐control) were purchased from GenePharma Co (Shanghai, China). SiRNAs were transfected into cells using Lipofectamine 3000 (Life Technologies, Carlsbad, CA, USA) according to the manufacturer's protocol. After 48 hrs, qRT‐PCR was used to measure the efficiency of siRNA knock‐down. The primer sequences were showed in S1.

For stable knock‐down of HIF‐2α shRNA (sh‐HIF‐2α) and scrambled control RNA (sh‐control) were inserted into the lentiviral vector (LV3H1/GFP&Puro vector). After transfection of 293T cells for 72 hrs, the viral supernatants were collected. Using a LentiX™ Concentrator overnight at 4°C (Clontech, Mountain View, CA, USA), lentiviral particles were concentrated and tittered to 10^9^ TU/ml (transfection unit/ml). Panc‐1 and Capan‐2 cells (5 × 10^5^ cells/well) were seeded in six‐well culture plates and cultured in DMEM with 10% FBS and infected with virus and polybrene 24 hrs later. Positive clones were screened with puromycin (2 μg/ml and 5 μg/ml, respectively) for 2‐3 weeks. The primer sequences were showed in S1.

### Boyden chamber cell invasion

Invasion assays were performed using the BD Biocoat Matrigel Invasion Chamber (8 μm; BD Biosciences, SanJose, CA, USA) following the manufacturer's instructions. After transduced with sh‐HIF‐2α or sh‐control, 1 × 10^4^ cells were plated in the upper chamber incubated in DMEM without FBS. The bottom chamber contained DMEM with 10% FBS to stimulate invasion. After incubation at 20% or 1% O_2_ for 36 hrs, the bottom chamber insert was stained with 0.1% crystal violet, and cells were counted and photographed with a Nikon microscope.

### Cell proliferation assays and colony formation assays

Cell counting kit‐8 (CCK‐8) assay was used to measure cell proliferation according to the manufacturer's instructions. Cells were seeded in 96‐wells plates (2 × 10^3^ cells/well) and cultured at 20% or 1% O_2;_ CCK‐8 solution (10 μl, Dojindo Molecular Technologies, Kyushu, Japan) was added at 0, 24, 48 and 72 hrs, respectively. Then, cells were incubated at 20% or 1% O_2_ for 4 hrs. Absorbance was measured at a wavelength of 450 nm using a microplate reader.

To examine HIF‐2α on cell growth in Panc‐1 and Capan‐2 under different levels of O_2_, 1000 sh‐HIF‐2α or sh‐control cells were seeded into the six‐well plates. After being incubated for 10–14 days at 37°C at 20% or 1% O_2,_ the plates were fixed with methanol, stained with 2% crystal violet and counted.

### Wound healing assay

Wound healing assay was conducted to examine the capacity of cell migration and invasion. When the cells reached 90–95% confluent, the wound was generated by scratching the surface of the plates with a 10‐ul pipette tip. Cells then were incubated in DMEM without FBS and then photographed with a Nikon microscope at 0 hr, 24 hrs, 48 hrs.

### Cell‐cycle analysis

Sh‐HIF‐2α or sh‐control cells were incubated at 20% or 1% O_2_ for 48 hrs. After incubated in cold ethyl alcohol at 4°C overnight, cells were analysed with flow cytometer (FACScan; BD Biosciences, Franklin Lake, NJ, USA) equipped with Cell Quest software (BD Biosciences, NJ, USA).

### Protein extraction and Western blot analysis

After washed with PBS, cells were then lysed with the mixture of RIPA buffer (Invitrogen) and protease inhibitor. And the supernatant was collected after centrifugation. A bicinchoninic acid protein assay kit (Pierce, Rockford, IL, USA) was used to calculate the protein concentration of each sample. Equal amount of proteins was separated on a SDS‐polyacrylamide gel electrophoresis and then transferred to polyvinylidene fluoride membranes. The membranes were blocked in 5% skim milk for 1 hr at room temperature and then incubated in the primary antibodies: rabbit anti‐human HIF‐2α antibody (1:1000, #NB100‐105, Novus, Colorado, USA), mouse anti‐human GOT1 (1:1000, #60317‐1‐Ig, proteintech, Rosemont, USA), rabbit anti‐human GOT2 antibody (1:1000, #14800‐1‐AP, proteintech, Rosemont, USA), rabbit anti‐human GLS1 antibody (1:600, #12855‐1‐AP, proteintech, Rosemont, USA), rabbit anti‐human GLUD1 antibody (1:1000, #ab168352, Abcam, Cambridge, MA), rabbit anti‐human ME1 antibody(1:1000, #ab97445, Abcam, Cambrigde, MA), mouse anti‐human MDH1 antibody(1:500, #ab76616, Cambrigde, MA) rabbit anti‐human β‐actin antibody (1:1000, #ab18162, Abcam, MA) at 4°C overnight. Horseradish peroxidase‐conjugated secondary antibodies (Cell Signaling Technology, Beverly, MA) and an ECL chemiluminescence kit (Pierce) were used to detect bound antibody.

### Immunohistochemistry and scoring analyses

Paraffin‐embedded samples of primary carcinomas were provided by Sun Yat‐Sen University (Guangdong, China). The xenograft tumour specimens from nude mice were fixed in 4% paraformaldehyde and then embedded in paraffin. The samples were deparaffinized in xylene and rehydrated in grade series of ethanol followed by heat‐induced epitope retrieval in citrate buffer (pH = 6.0). Antigen retrieval was carried out in 10 mmol/l citrate buffer (pH = 6.0) in a microwave oven for 15 min.; 3% hydrogen peroxide was used to exhaust the activity of endogenous peroxidase for 10 min. at room temperature. Rabbit anti‐human HIF‐2α antibody (1:300, #NB100‐105, Novus), rabbit anti‐Ki‐67(1:125, Abcam, Cambridge, MA) was applied over night at 4°C. After sufficient phosphate‐buffered saline washed, sections were stained with corresponding secondary antibody for 30 min. at room temperature. The sections were incubated with streptavidin‐HRP conjugate complex for 30 min. at 37°C. The slides were developed with 3, 3′‐diaminobenzidine. Sections were counterstained with haematoxylin.

**Figure 1:**
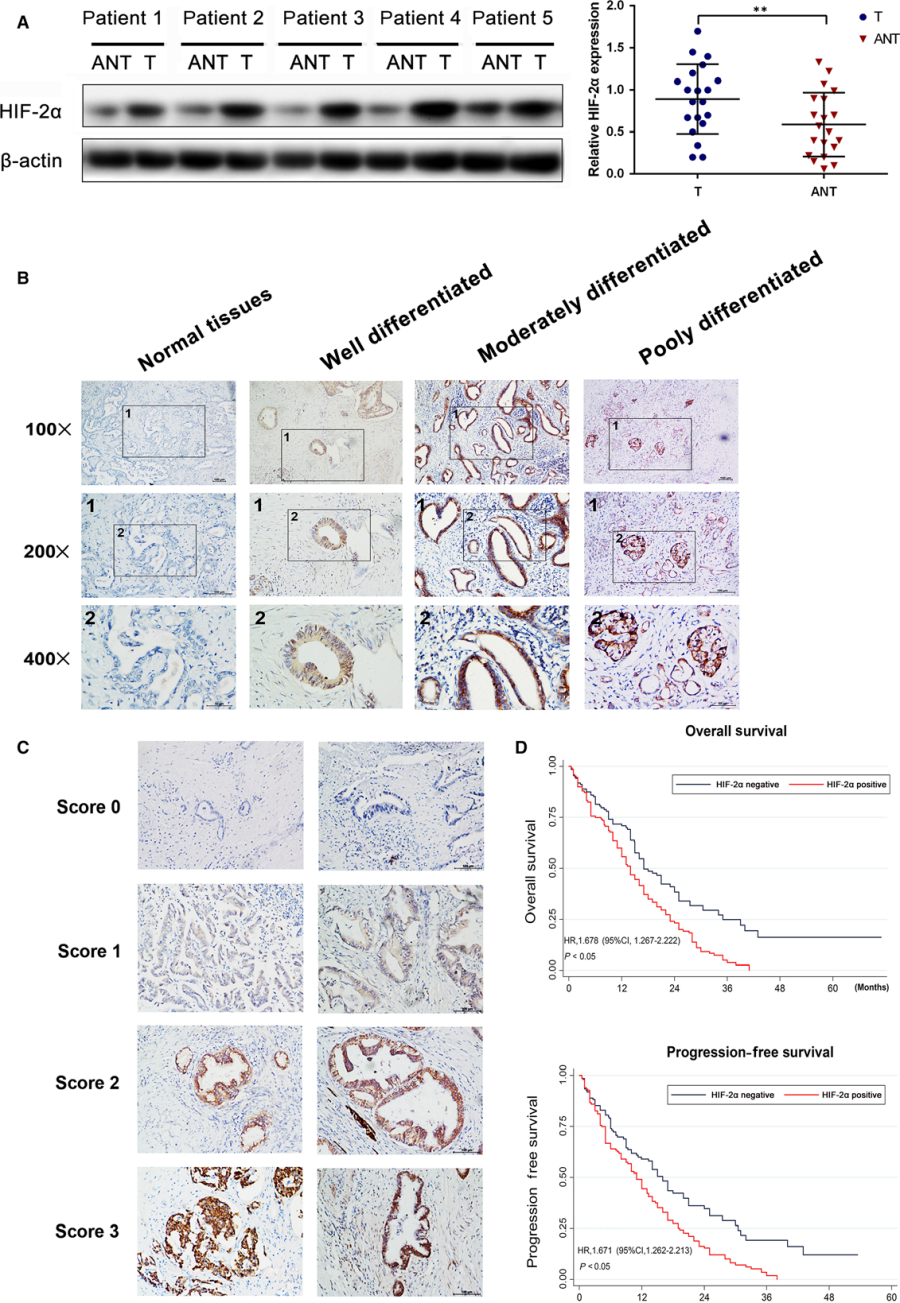
Up‐regulation of HIF‐2α correlated with poor prognosis in human PDAC **.** (**A**) Western blot analysis and qRT‐PCR analysis of HIF‐2α expression in PDAC tissues (T) and matched adjacent non‐tumour tissues (N). The mRNA and protein levels were normalized to β‐actin. **P < 0.01 (**B**) The images of immunohistochemistry for each differentiation degree in paraffin‐embedded PDAC and non‐tumours tissues. (**C**) The representative images for each score were showed. (**D**) The relationship between the expression of HIF‐2α and OS or PFS.

HIF‐2α expression in the PDAC specimens was blind‐quantified by two pathologists using a previously described scoring system. Briefly, the immunostaining intensity of each sample was graded as negative = 0, weak = 1, moderate = 2 or strong = 3 (Fig. 1C). The proportion of positively staining cells was assessed as the percentage. The score was then calculated as the intensity score multiplied by the percentage of cells stained (score = intensity x % of positive cells). The samples were classed as low (score < 140) or high (score ≥ 140) HIF‐2α expression. Imageswere visualized using a Nikon ECLIPSE Ti (Fukasawa, Japan) microscope system and processed with Nikon software.

### Glutamine consumption

Panc‐1 and Capan‐2 were treated in 3 ml DMEM containing 2.5 mmol/l glutamine in six‐well plates, 1 × 10^6^ cells were seeded in each well and mitomycin was used to inhibition proliferation. Every 12 hrs replaced the medium, testing the remaining medium volume and the concentration of glutamine in them. The effect of HIF‐2α on glutamine consumption was measured after cells were treated at 20%, 1% O_2_ for 48 hrs.

### NADP^+^/NADPH ratio measurement

NADP^+^/NADPH ratios were measured using the NADP^+^/NADPH assay kit (Abcam, ab65349) according to the manufacturer's instructions. Briefly, 10^5^ cells were collected on ice in extraction buffer by performing two freeze/thaw cycles (20 min. on dry ice followed by 10 min. at 37°C) and concentration was obtained by comparison with standard curves.

### Measurement of intracellular ROS levels

Intracellular ROS was measured using the ROS detection kit (Beyotime Company, S0033, Shang Hai, China) according to the manufacturer's instructions. Briefly, after treatment at 1% O_2_ or 20% O_2_ for 48 hrs, cells were washed twice in phosphate‐buffered saline (PBS) and then incubated with 10 μmol/l DCFH‐DA at 37°C for 20 min. Then, DCF fluorescence distribution of 2 × 10^4^ cells was detected by fluorescence microplate reader at an excitation wavelength of 488 nm and at an emission wavelength of 525 nm.

### **Tumo**u**r formation assay**

The animal care and experimental protocols were approved by the institutional guidelines of Guangdong Province and by the Use Committee for Animal Care. BALB/c nude mice (4–6 weeks old) were subcutaneously injected the left flank with the Panc‐1 cells expression the control shRNA and the right flank with the Panc‐1 cells expression the shRNA to HIF‐2α (7 × 10^6^ cells/flank). The volume of each tumour was measured every 3 days. Tumour volume was calculated using the following formula: $\text{volume}=\left(L\times W^{2}\right)/2$ , where *L* and *W* are the longest and shortest diameters, respectively. On the 21th day after injection, mice were killed and tumours were harvested and photographed.

### Statistical analysis

Data are presented as the mean ± S.D. of three independent experiments. Cumulative survival time was calculated using the Kaplan–Meier method and analysed by the log‐rank test. A multivariate Cox proportional hazards model was used to estimate the adjusted hazard ratios and 95% confidence intervals and to identify independent prognostic factors. All statistical analyses were performed with SPSS19.0 (SPSS Inc, Chicago, IL, USA). Statistical evaluation of data was performed using one‐way anova for multiple groups, Student's *t*‐tests for two groups and chi‐square test (χ^2^ test) for non‐parametric variables. The threshold for statistical significance was set at *P < 0.05, **P < 0.01.

## Results

### HIF‐2α expression is increased in PDAC and associated with poor prognosis in human PDAC patients

To evaluate the expression of HIF‐2α in PDAC and non‐tumorous tissues, we first utilized qRT‐PCR and Western blot analysis on the cases of PDAC. HIF‐2α expression was increased in all of the cases as compared to the adjacent normal tissues (Fig. 1A). Moreover, we used immunohistochemistry analysis to measure the HIF‐2α expression in 283 PDAC tissues and 283 paired normal tissues (Fig. 1B). HIF‐2α was significantly overexpressed in PDAC tissues as compared with normal tissues and obviously higher in poorly differentiated tissues as compared to well‐differentiated tissues (Fig. 1B). Clinicopathological correlation analysis revealed positive correlation between elevated HIF‐2α levels with TNM stage, lymph‐node metastasis, differentiation and perineural invasion (Table 1).

Next, we examined the correlation between expression of HIF‐2α and overall survival (OS) or progression‐free survival (PFS). Kaplan–Meier survival analysis noted that HIF‐2α overexpression was correlated with reduced OS (HR, 1.678; 95% CI: 1.267–2.222, *P* < 0.05) and PFS (HR, 1.671; 95% CI: 1.262–2.213, *P* < 0.05) (Fig. 1D). To further evaluate the prognostic factors associated with OS and PFS in PDAC, we first carried out multivariate Cox regression analyses using gender, age, differentiation, TNM stage, lymph‐node metastasis, perineural invasion and HIF‐2α level expression as parameters. The results showed that high HIF‐2α expression was an independent predictor of OS and PFS in PDAC patients (Table 2). These findings clearly demonstrated that HIF‐2α might be a key factor for poor prognosis of PDAC and associated with positive lymph‐node metastasis and TNM stage.

**Table 2:** Multivariate Cox regression analyses of OS and PFS in PDAC patients

Variables	Hazard Ratio	Std. Err.	z	*P* > z	95% Conf. Interval	
Overall survival						
Gender	0.819069	0.118835	−1.38	0.169	0.616344	1.088473
Age	1.085215	0.164879	0.54	0.59	0.805732	1.461641
Differentiation	0.861365	0.083491	−1.54	0.124	0.71233	1.04158
TNM stage (AJCC)	1.962722	0.359883	3.68	0.003**	1.370197	2.811476
Lymph‐node metastasis	1.259477	0.20096	1.45	0.148	0.921242	1.721895
Perineural invasion	0.584926	0.222848	−1.41	0.159	0.277208	1.23423
HIF‐2α level	1.418071	0.217761	2.27	0.023*	1.049509	1.916061
Progression‐free survival						
Gender	0.912942	0.131993	−0.63	0.529	0.687664	1.212021
Age	0.874395	0.134805	−0.87	0.384	0.646366	1.182869
Differentiation	0.840913	0.081486	−1.79	0.074	0.695454	1.016796
TNM stage (AJCC)	1.79714	0.333591	3.16	0.002**	1.249047	2.58574
Lymph‐node metastasis	1.417982	0.225453	2.2	0.028*	1.038325	1.936459
Perineural invasion	0.724537	0.280056	−0.83	0.404	0.339661	1.545525
HIF‐2α level	1.358757	0.208601	2	0.046*	1.005685	1.835783

*P < 0.05, **P < 0.01.

### HIF‐2α knock‐down impairs cell growth, migration and invasion of PDAC cells *in vitro*

**Figure 2:**
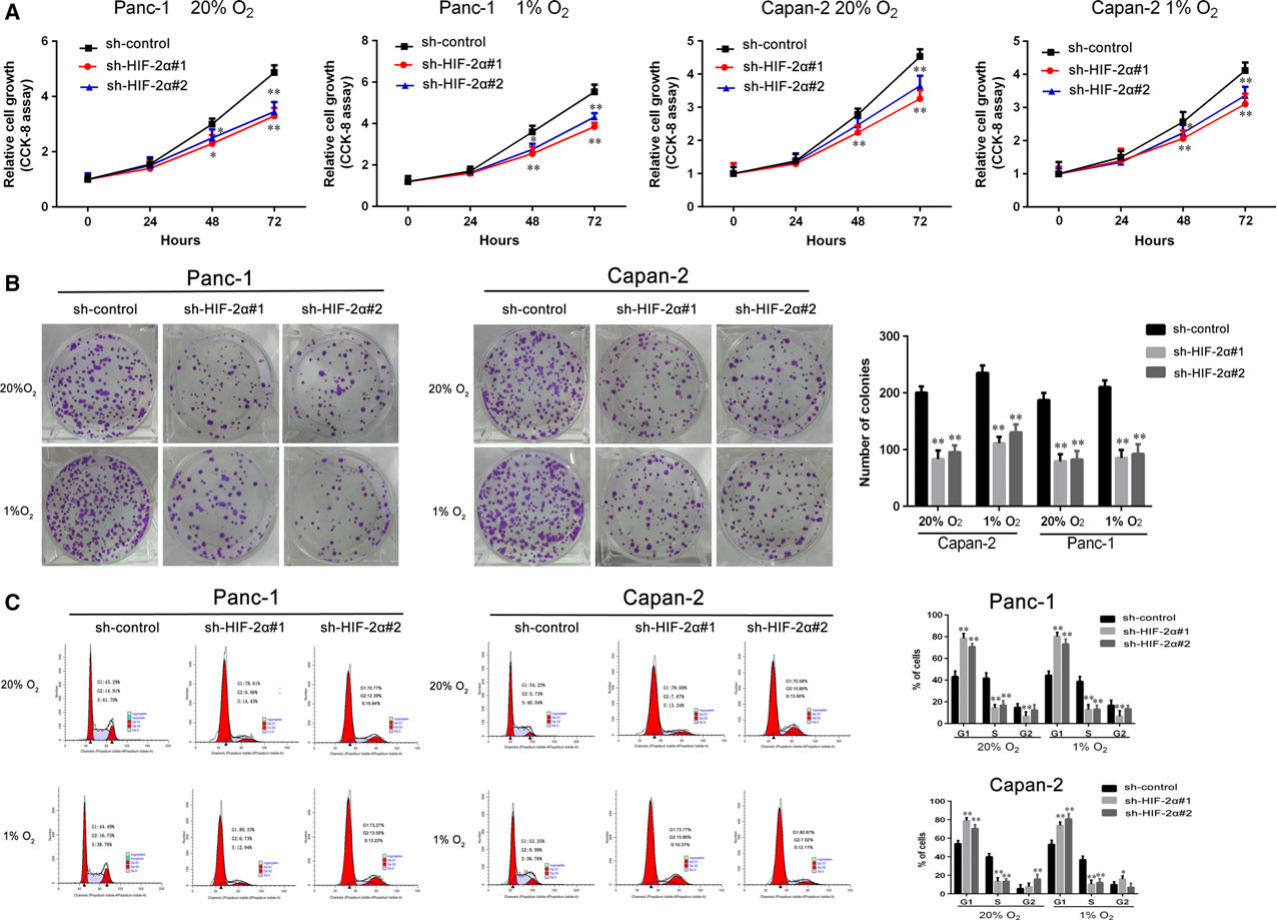
HIF‐2α knock‐down impairs cell growth of PDAC cells *in vitro*. (**A**) Sh‐HIF‐2α‐transduced Panc‐1 and Capan‐2 cultured at 1% or 20% O_2_. The cell viability measured by CCK‐8 assay. (**B**) After being incubated at 1% or 20% O_2_, the proliferation of sh‐HIF‐2α‐transduced Panc‐1 and Capan‐2 by colony formation assay. (**C**) Capan‐2 and Panc‐1 cells were stably transfected by sh‐HIF‐2α and cultured at 1% or 20% O_2_ for 48 hrs. The cell‐cycle distribution was assessed by flow cytometry. Data represent the mean ± S.D. from three independent experiments. *P < 0.05, **P < 0.01.

Panc‐1 and Capan‐2 cells were transfected with siRNA targeted HIF‐2α or control siRNA, we tested the transfection efficiency of HIF‐2α#1 and HIF‐2α#2 (Fig. S1). We then stably suppressed HIF‐2α in Panc‐1 and Capan‐2 cells by lentiviral transfection. CCK‐8 assays revealed that there was a decrease of cell growth rate in sh‐HIF‐2α group than sh‐control group in Panc‐1 and Capan‐2 cells (Fig. 2A). Consistent with our cell growth data, HIF‐2α knock‐down greatly attenuated the colony numbers of Panc‐1 and Capan‐2 cells (Fig. 2B). Furthermore, to further investigate the growth inhibition, we compared the cell‐cycle profiles of HIF‐2α down‐regulation cells with controls by flow cytometry. As shown in Figure 2C, HIF‐2α knock‐down dramatically increased the cell population in the G0/G1 phase, whereas it reduced the cell population in the S phases. Together, these data indicated the critical role of HIF‐2α on growth of PDAC cells.

**Figure 3:**
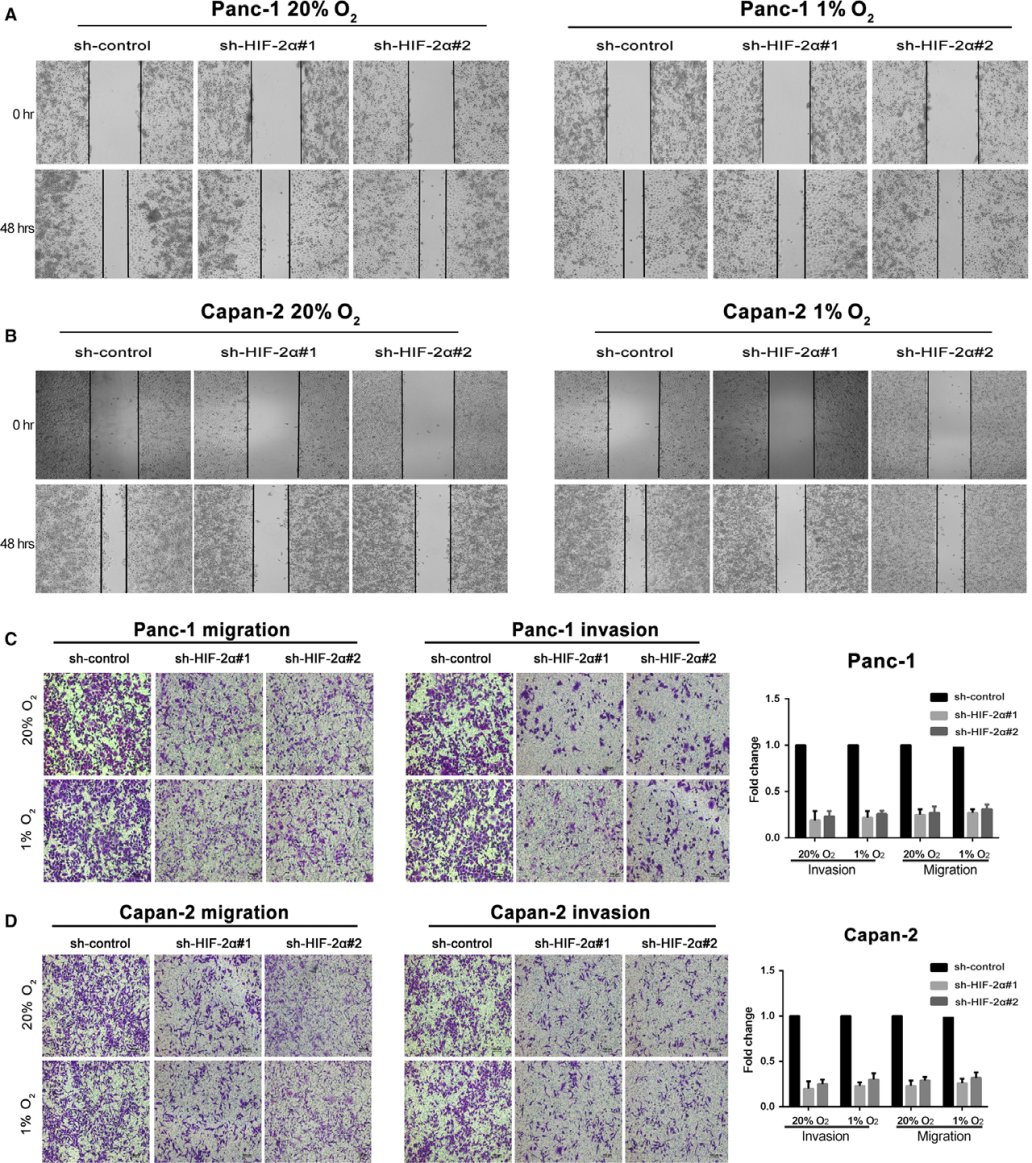
HIF‐2α knock‐down impairs migration and invasion of PDAC cells *in vitro*. (**A** and **B**) The motility of Panc‐1 and Capan‐2 cells transduced with sh‐HIF‐2α when compared with the controls by the wound healing assay. (**C** and **D**) The migration and invasion of Panc‐1 and Capan‐2 cells stably transfected with sh‐HIF‐2α when compared with the controls by transwell assays. Cells were respectively incubated at 20% or 1% O_2._ Values represented the mean ± S.D. from three independent experiments. *P < 0.05, **P < 0.01.

Migration and invasion are crucial for cancer progression and result in poor prognosis. We performed wound healing assay to indicate that knock‐down of HIF‐2α in Capan‐2 and Panc‐1 cells dramatically attenuated cell motility (Fig. 3A and B). Moreover, transwell assay was utilized to show that HIF‐2α knock‐down decreased the migration and invasion in PDAC cells (Fig. 3C and D). These data demonstrated that HIF‐2α plays a critical role on the migration and invasion of PDAC cells.

### HIF‐2α regulates non‐canonical glutamine metabolism in chronic hypoxic conditions

**Figure 4:**
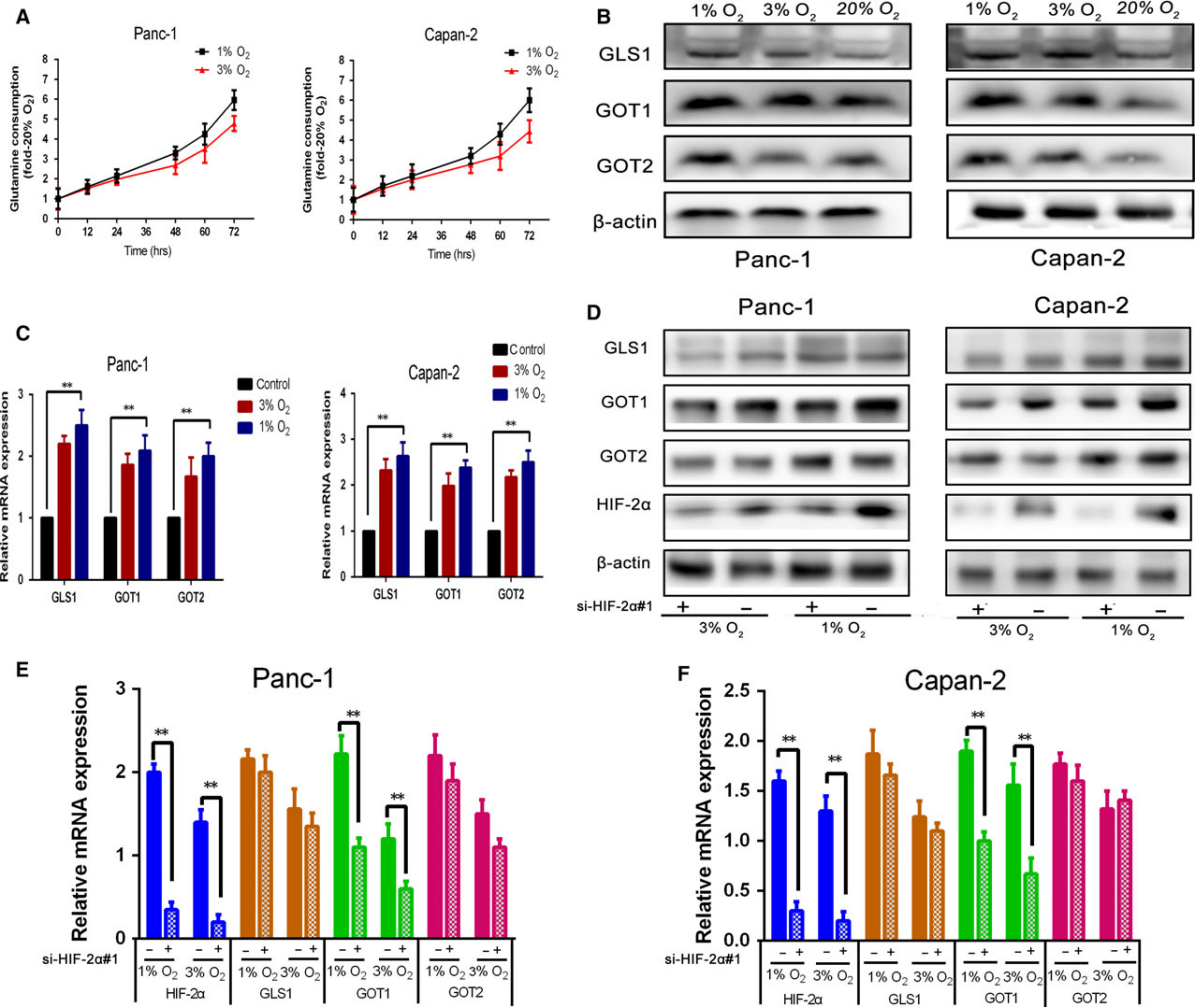
Prolonged hypoxia increases glutamine metabolism in PDAC cells, and HIF‐2α can promote non‐canonical glutamine metabolism in chronic hypoxic conditions. (**A**) Time course of glutamine consumption at 1%, 3% or 20% O_2_, each time data point is an average of triplicate experiments. (**B**) Panc‐1 and Capan‐2 were incubated for 48 hrs at 1%, 3% or 20% O_2_, GLS1, GOT1 and GOT2 protein were measured by Western blot. β‐Actin was used as loading control. (**C**) Panc‐1 and Capan‐2 were incubated for 48 hrs at 1%, 3% or 20% O_2_, GLS1, GOT1 and GOT2 mRNA were measured by qRT‐PCR. Data are presented as mean ± S.D. from three independent experiments. (**D** and **F**) Si‐HIF‐2α‐transfected Panc‐1 and Capan‐2 cultured at 1% or 3%O_2_ for 48 hrs. The level of HIF‐2α and glutamine metabolism enzymes mRNA and protein were determined by qRT‐PCR (mRNA) and Western blot (protein), and β‐actin was used as loading control. Data are presented as mean ± S.D. from three independent experiments. *P < 0.05, **P < 0.01.

To investigate whether hypoxia could affect the metabolism of glutamine in PDAC cells, we measured the glutamine consumption rate. As shown in Figure 4A, with the reducing oxygen concentration, Panc‐1 and Capan‐2 depleting glutamine rates were increasing, especially after 48 hrs. Moreover, we evaluated the levels of mRNA and protein of glutamine metabolism enzymes by qRT‐PCR and Western blot analysis, respectively (Fig. 4B and C). These results showed that glutamine metabolism is increased under the reduced oxygen concentration. Next, we examined the effect of HIF‐2α on glutamine metabolism in PDAC cells. Si‐HIF‐2α‐transfected cells were separately incubated at 1% O_2_ or 20% O_2_ for 48 hrs. The glutamine consumption, intracellular ROS levels and NADP^+^/NADPH ratio were showed in Figure S2 A and B. These results demonstrated that HIF‐2α knock‐down significantly attenuated glutamine consumption rates, increased ROS levels and NADP^+^/NADPH ratios. We measured the expression of GLS1, GOT1, GOT2 related mRNA and protein after down‐regulating HIF‐ 2α by qRT‐ PCR and western blot analysis. As shown in Figure 4D‐ F, HIF‐ 2α knock‐ down induced a significantly decrease expression of GOT1, but had no effect on the expression of GLS1 and GOT2. Si‐HIF‐2α‐transfected Panc‐1 and Capan‐2 were separately incubated at 1% O_2_ for 48 hrs. Other levels of glutamine metabolism enzymes were tested by qRT‐PCR and Western blot analysis. We found that HIF‐2α had no effect on the expressions of GLUD1, MDH1, ME1 (Fig. S2C and D). Taken together, our results showed that HIF‐2α could promote non‐canonical glutamine metabolism in hypoxic conditions.

### HIF‐2α knock‐down suppresses tumorigenicity and non‐canonical glutamine metabolism of PDAC cells *in vivo*

**Figure 5:**
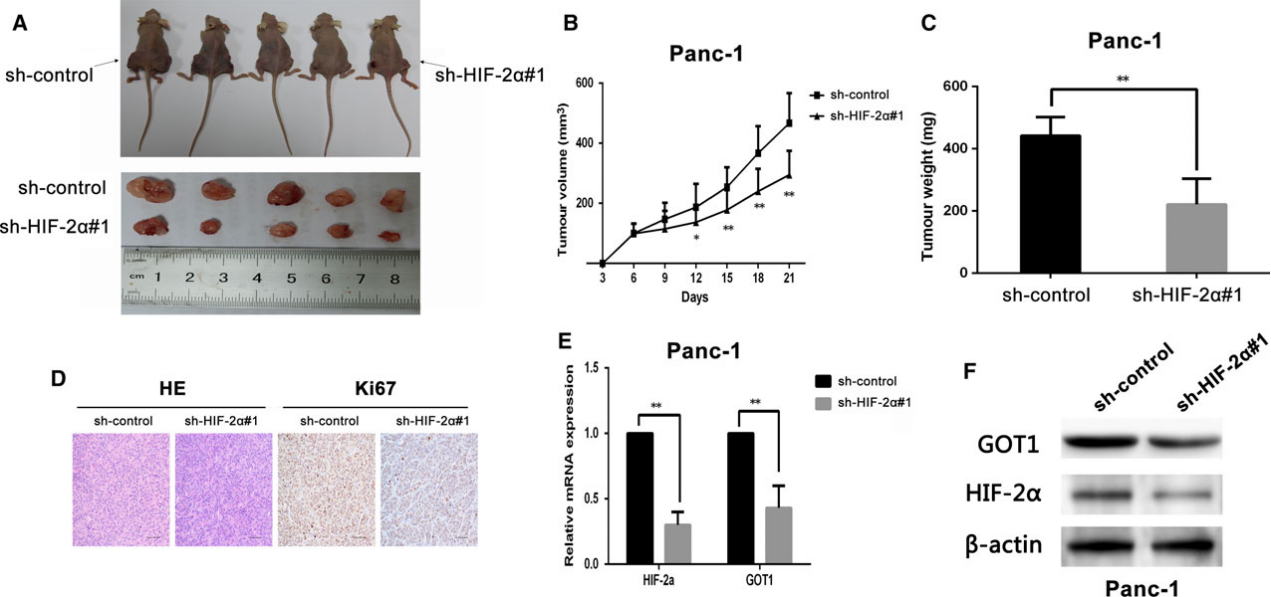
HIF‐2α depletion counteracts tumour growth and non‐canonical glutamine metabolism *in vivo*. (**A**) Representative images of tumour‐bearing mice and tumours removed from the mice. (**B**) Tumour volumes were measured on the indicated days. (**C**) Tumour weights were shown as the means ± S.D. when the tumours were harvested. (**D**) Representative images (×200) of H&E and IHC staining of the tumours. Ki67 was used as proliferation index. (**E** and **F**) qRT‐PCR and Western blot analysed the expression of HIF‐2α and GOT1 in tumour tissues from sh‐HIF‐2α Panc‐1 group compared with control group, *P < 0.05, **P < 0.01.

To further explore the promotion of HIF‐2α in tumour progression and non‐canonical glutamine metabolism, the HIF‐2α stable knock‐down or sh‐control cells were subcutaneously injected into nude mice. We measured the tumour growth activity and showed that tumour growth in the sh‐HIF‐2α group was significantly decreased compared with the sh‐control group (Fig. 5A). The size and weight of tumours from the HIF‐2α knock‐down group were significantly lower than those of tumours from the sh‐control group (Fig. 5B and C). Immunostaining revealed that knock‐down HIF‐2α could decrease the positive rate of Ki67 compared with the control group (Fig. 5D). Furthermore, we measured the inhibition of HIF‐2α and GOT1 in the xenotransplanted tumours derived from the sh‐HIF‐2α and sh‐control groups, respectively. The results noted that sh‐HIF‐2α groups had lower expression of GOT1 expression than the sh‐control group (Fig. 5E and F). Taken together, our results demonstrated that HIF‐2α knock‐down suppressed tumorigenicity and non‐canonical glutamine metabolism of PDAC cells *in vivo*.

### HIF‐2α regulates non‐canonical glutamine metabolism *via* activation of PI3K/mTORC2 pathway

**Figure 6:**
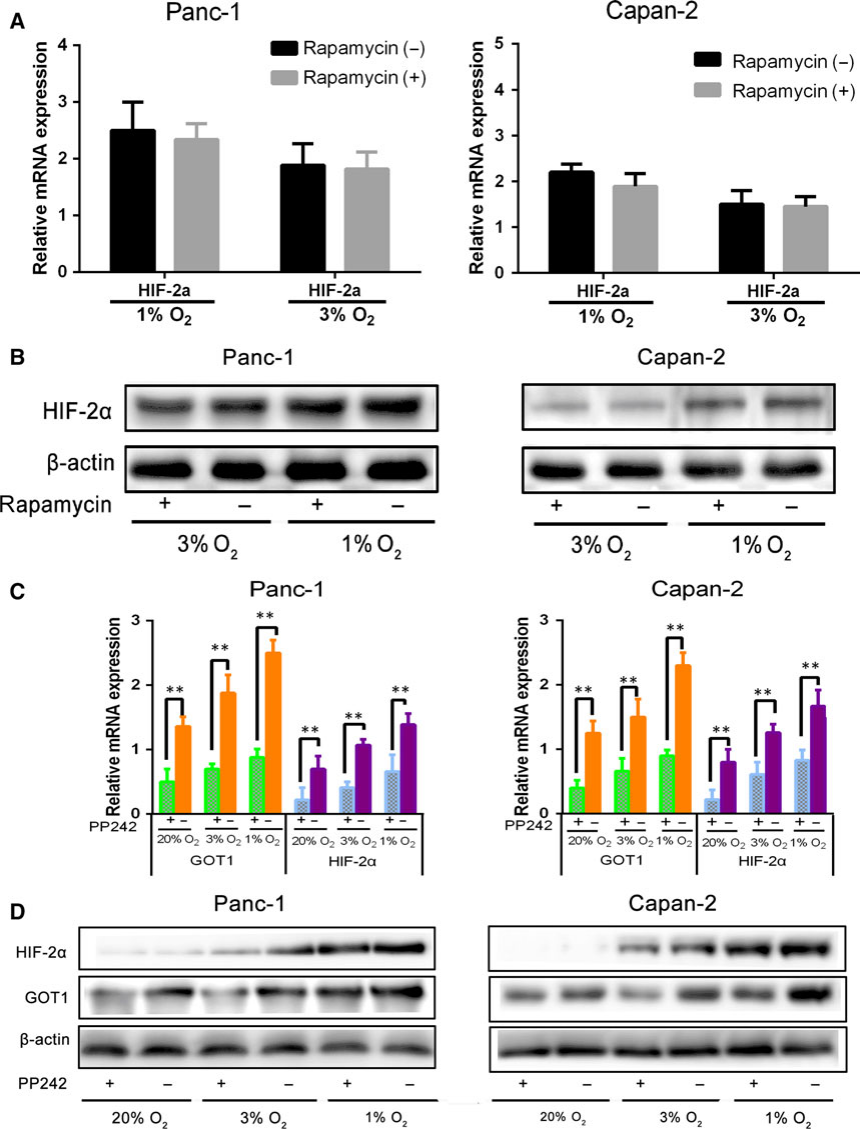
mTORC2 not mTORC1 regulates HIF‐2α, GOT1 in PDAC under prolonged hypoxia. (**A** and **B**) HIF‐2α mRNA and protein levels determined by qRT‐PCR (mRNA) and Western blot (protein) after treatment of Panc‐1 and Capan‐2 cells with mTORC1 inhibitor rapamycin for 48 hrs at 3% or 1% O_2_. β‐Actin was used as loading control. (**C** and **D**) Panc‐1 and Capan‐2 cells were treated with mTORC1/mTORC2 inhibitor PP242 and cultured for 48 hrs at 20%, 3% or 1% O_2,_ and HIF‐2α, GOT1 mRNA and protein levels were determined by qRT‐PCR (mRNA) and Western blot (protein). β‐Actin was used as loading control. Data are presented as mean ± S.D. from three independent experiments. *P < 0.05, **P < 0.01.

The phosphatidylinositol 3‐kinase (PI3K)/AKT/mTOR pathway plays a significantly role in pancreas accelerating the formation of PDAC, activating in premalignant pancreatic lesions. Previous studies demonstrated that mTORC2 regulates the transcription levels of HIF‐2α in multiply cancer 15, 17, 18, 19. To explore whether PI3K/mTOCR2 plays an important role in regulating HIF‐2α and non‐canonical glutamine metabolism in PDAC, HIF‐2α and GOT1 mRNA and protein levels were measured after the pharmacologic inhibitions suppressed the activity of target proteins. Panc‐1 and Capan‐2 were exposed to rapamycin for 48 hrs at 1%, 3% O_2_. As shown in Fig. 6A and B, inhibiting mTORC1 did not have significant influence on HIF‐2α expression. Interestingly, our results noted the positive correlation between mTORC2 and HIF‐2α (Fig. 6C and D). Panc‐1 and Capan‐2 were treated with dual mTORC1/mTORC2 inhibitor PP242 for 48 hrs at 20%, 1%, 3% O_2_. HIF‐2α, GOT1 mRNA expression and protein were determined by qRT‐PCR and Western blot, respectively. The results demonstrated that inhibition of mTOR2 activity by PP242 down‐regulated the expression of HIF‐2α and GOT1 in mRNA and protein levels (Fig. 6C and D). GDC‐0068 is a highly selective pan‐AKT inhibitor. Panc‐1 and Capan‐2 were exposed to GDC‐0068 for 48 hrs at 1%, 3% O_2_. As shown in Figure S3, inhibiting AKT has no effect on HIF‐2α expression. Moreover, we found pharmacologic inhibition of PI3K activity with LY294002 attenuated the expression of HIF‐2α and GOT1 (Fig. S4). Taken together, these data indicated that HIF‐2α regulates non‐canonical glutamine metabolism by activation of PI3K/mTORC2 pathway.

## Discussion

PDAC is a fatal and poor prognostic gastrointestinal carcinoma 1. Metabolic Reprogramming is an important feature of the tumour environment 6. The recovery of non‐canonical pathway of glutamine is a hallmarks progression 6. Pancreatic cancers are clinically and histologically characterized as hypovascular tumours 28, 29. They both play critical role in development and progression of pancreatic cancer. Identification the relationship between non‐canonical pathway of glutamine and HIFs is crucial for the management of PDAC, and the pathway regulate non‐canonical glutamine metabolism in hypoxia may provide avenues for therapeutic intervention.

Numerous and severe hypoxic regions in PDAC were verified to be related with progression aggressiveness and poor prognosis 30. HIFs are important in hypoxia that can transcript many genes to code protein and play a role in many aspects of cancer biology 31. HIF‐1α and HIF‐2α protein were detected in most types of human tumours, including pancreatic, bladder, colon, breast, glial, hepatocellular, prostate and renal tumours 23. In our study, we validated that HIF‐2α was overexpressed in PDAC tissues and was associated with TNM stage, lymph‐node metastasis, differentiation and perineural invasion of PDAC individuals. HIF‐2α was a potential target of poor prognosis which was also revealed from worse overall survival and progression‐free survival.

After analysing with clinical samples, we utilized PDAC cells to search for further support. We found that knock‐down of HIF‐2α led to decrease in proliferation and antagonistic effects on cell‐cycle progression of PDAC cells. Moreover, the similar effect on growth of HIF‐2α was also demonstrated *in vivo*. Metastasis plays a crucial role on tumour progression. We revealed that knock‐down of HIF‐2α can repress migration and invasion of PDAC cells. Despite that the effect of HIF‐2α on tumour metastasis *in vivo* was not observed in our trial, we demonstrated that lymph‐node metastasis was connected with high level of HIF‐2α in PDAC tissues.

To meet the demands of energy and biosynthesis under hypoxia, tumour cells must develop metabolic responses like high glycolytic rate and hexosamine biosynthetic pathway activation 27. Our results demonstrated that in PDAC cell lines, the consumption of glutamine was increased under reduced hypoxia concentration, especially in prolonged hypoxia. Meanwhile the key enzyme expression of glutamine metabolism pathway was also increased.

Considering HIFs were important factors in hypoxia environment, we suggested that HIF‐2α could regulate glutamine metabolism in prolonged hypoxia. As expected, after HIF‐2α was knocked down, the consumption of glutamine was reduced, while the intracellular ROS level and NADP^+^/NADPH ratio were increased compared with sh‐control group. The expression of GOT1 was inhibited by HIF‐2α knock‐down, which is a key enzyme of non‐canonical pathway of glutamine. The similar phenomenon was demonstrated *in vivo*. This is the first time to show HIF‐2α could regulate non‐canonical pathway of glutamine, which is a typical pathway of metabolism in PDAC. Our preliminary work revealed that tumour expression of GOT1 was significantly linked with tumour dedifferentiation, vascular invasion and lymphatic invasion 32. We revealed HIF‐2α was one of the upstream genes of GOT1 and regulate GOT1 to impact PDAC malignancy behaviours.

We future explored a potential pathway to regulate GOT1 *via* HIF‐2α. Nearly 100% of pancreatic adenocarcinomas have a K‐RAS mutation 33, 34. Activated K‐RAS promotes the PI3K signalling pathway and the serine/threonine kinase mTOR 35. It has been verified the reprogramming of glutamine metabolism is mediated by oncogenic KRAS 6. In our study, we demonstrated that pharmacologic inhibition of PI3K or mTORC2 can repress HIF‐2α transcription and down‐regulate GOT1. In contrast, the expression of HIF‐2α was independent of mTORC1 or AKT. It revealed that PI3K/mTORC2 pathway played a significant role in non‐canonical glutamine metabolism of PDAC *via* HIF‐2α.

In conclusion, we determined that HIF‐2α is an independent prognostic factor for PDAC and promotes the progression of PDAC both *in vitro* and *in vivo*. In prolonged hypoxia environment, HIF‐2α can regulate non‐canonical pathway of glutamine, and PI3K/mTORC2 is one of the pathways to regulate HIF‐2α to promote non‐canonical glutamine metabolism. Moreover, we wondered whether HIF‐2α might be a potential target for the treatment of PDAC.

## Conflicts of interest

No potential conflicts of interest were disclosed.

## Author's contribution

L.WZ, C.CH and Z.XH contributed equally to this manuscript and carried out most of the experimental work; W.LS, and N.TW conducted the *in vitro* experiments; F.ZQ conducted the animal experiments; Z.Y.and P.WW conducted the IHC analysis; Y.HL and Z.SY conducted the glutamine consumption; L.ZH. and C.RF supervised the project and wrote the manuscript.

## Supporting information


**Figure S1** The efficiency of si‐HIF‐2α on Panc‐1 and Capan‐2 cells
**Figure S2** The effect of HIF‐2α on glutamine metabolism of PDAC cells in chronic hypoxic conditions.
**Figure S3** AKT takes no effect on the regulations of HIF‐2α and non‐canonical glutamine metabolism of PDAC.
**Figure S4** PI3K regulates HIF‐2α and non‐canonical glutamine metabolism of PDAC in prolonged hypoxia.
**Table S1** Primer of experiments.
