# Supplementary material for: HIF‐2α regulates non‐canonical glutamine metabolism via activation of PI3K/mTORC2 pathway in human pancreatic ductal adenocarcinoma
Source: J Cell Mol Med. 2017 May 24;21(11):2896–908. doi: 10.1111/jcmm.13202 (PMC5661146; doi:10.1111/jcmm.13202)
Supplement: Supplementary file 1 — Figure S1 The efficiency of si‐HIF‐2α on Panc‐1 and Capan‐2 cells Figure S2 The effect of HIF‐2α on glutamine metabolism of PDAC cells in chronic hypoxic conditions. Figure S3 AKT takes no effect on the regulations of HIF‐2α and non‐canonical glutamine metabolism of PDAC. Figure S4 PI3K regulates HIF‐2α and non‐canonical glutamine metabolism of PDAC in prolonged hypoxia. Table S1 Primer of experiments. [file JCMM-21-2896-s001.doc]

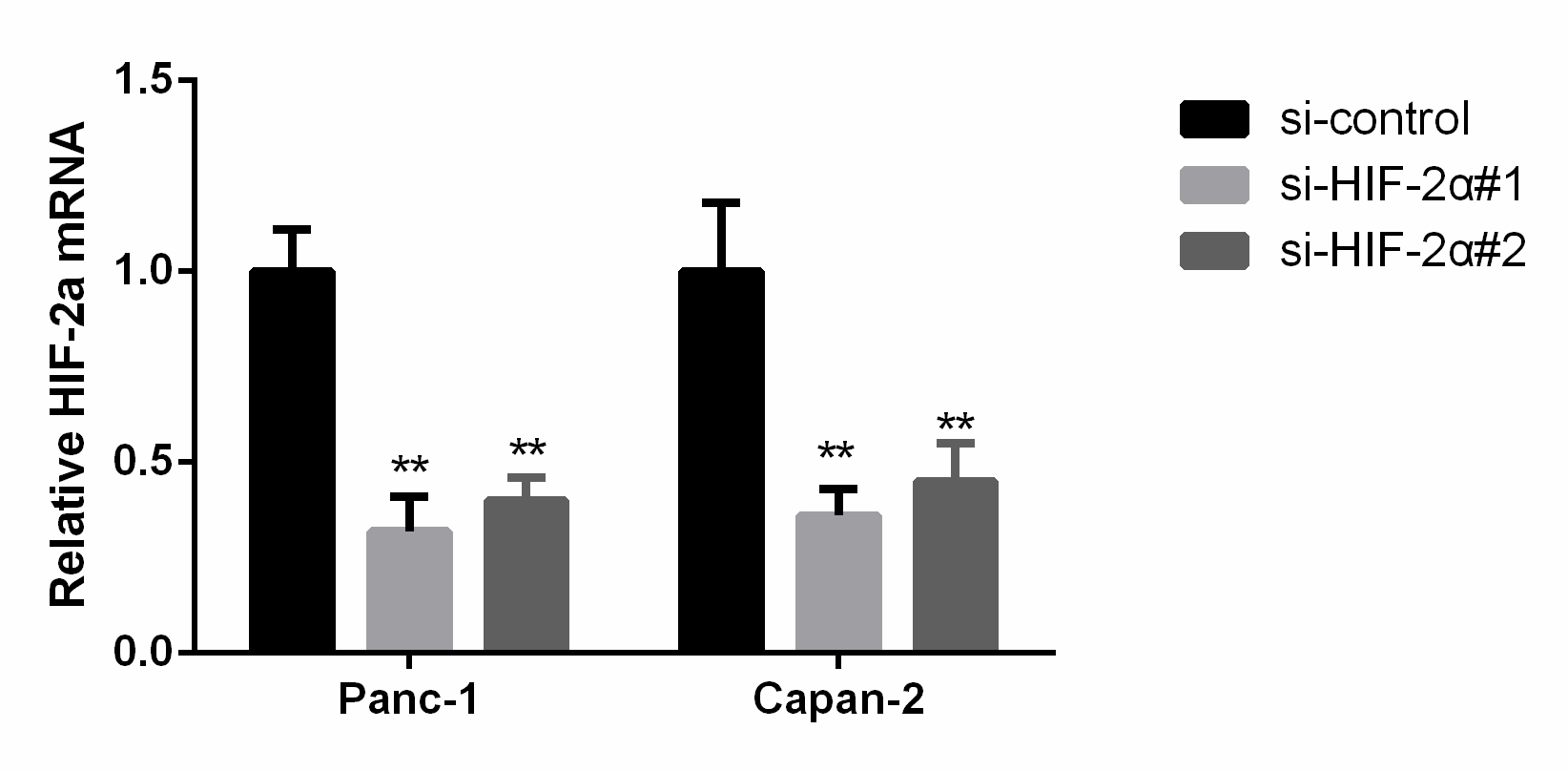
**Supplementary data**

**Supplementary Figure 1: The efficiency of si-HIF-2α on Panc-1 and Capan-2 cells**

**
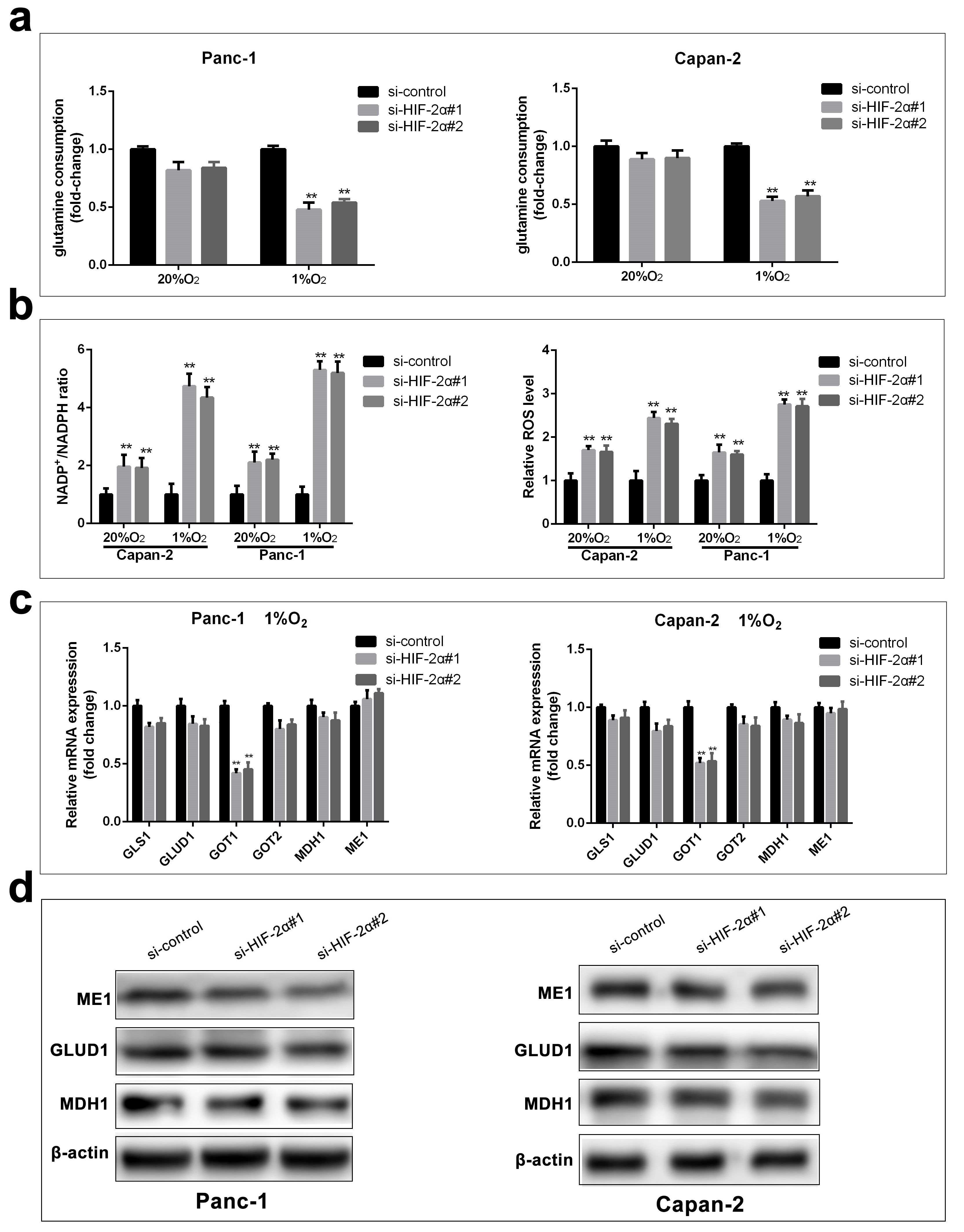
**

**Supplementary Figure 2: The effect of HIF-2α on glutamine metabolism of PDAC cells in chronic hypoxic conditions.** (a) The metabolism consumption of Panc-1 and Capan-2 cells expression two independent siRNA targeting HIF-2α after treated at 20% or 1%O_2_ for 48 h. (b) The NADP^+^/NADPH ratio and ROS levels in Panc-1 and Capan-2 cells expression a control siRNA or two independent siRNA targeting HIF-2α after treated at 20% or 1% O_2_ for 48 hr. (c-d) The other glutamine metabolism enzymes expression were determined by qRT-PCR (mRNA) and western blot (protein). β-actin was used as loading control. Data are presented as mean ± SD from three independent experiments. **p* < 0.05, ***p* < 0.01.


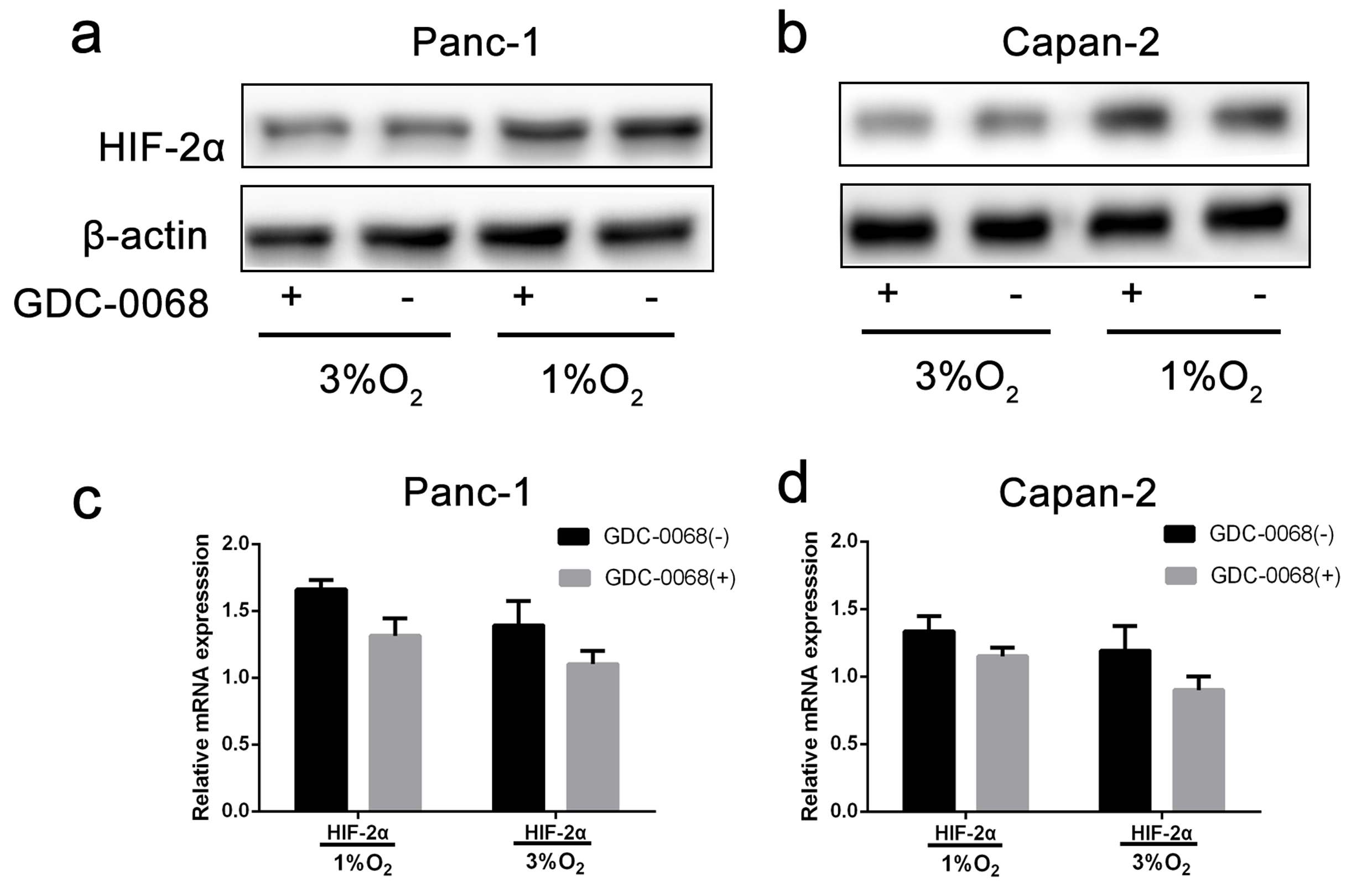


**Supplementary Figure 3: AKT takes no effect on the regulations of HIF-2α and non-canonical glutamine metabolism of PDAC.** (a-b) Panc-1 and Capan-2 cells were treated with pan-AKT inhibitor GDC-0068, and cultured for 48 hr at 3% or 1% O_2_. The expression level of HIF-2α was determined by qRT-PCR (mRNA) and western blot (protein). β-actin was used as normal control. Data are presented as mean ± SD from three independent experiments.


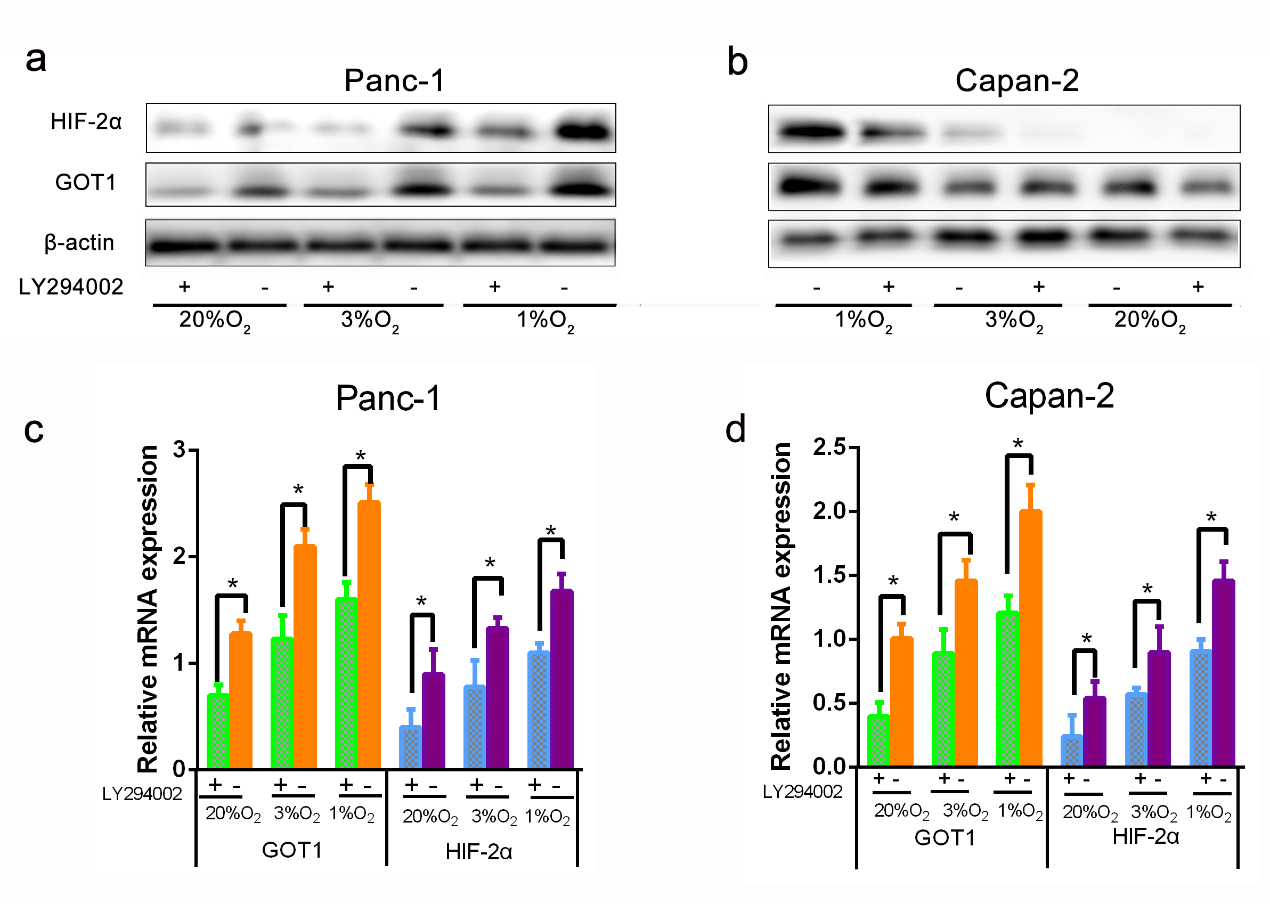


**Supplementary Figure 4: PI3K regulates HIF-2α and non-canonical glutamine metabolism of PDAC in prolonged hypoxia.** (a-b) Panc-1 and Capan-2 cells were treated with PI3K inhibitor LY294002, and cultured for 48 hr at 20%, 3% or 1% O_2_, The expression levels of HIF-2α and GOT1 were determined by qRT-PCR (mRNA) and western blot (protein). β-actin was used as normal control. Data are presented as mean ± SD from three independent experiments. **p* <0.05.

**Supplementary Table 1. Primer of experiments**

| Primer name | Nucleotide Sequence (5’-3’) | Application |
| --- | --- | --- |
| HIF-2α F | AAACGAGTCCGAAGCCGAA | qRT-PCR |
| HIF-2α R | CTCCACCTGTGTAAGTCCCATGA | — |
| GLUD1 F | AAGGGATTCTAACTACCACTTGCTC | — |
| GLUD1 R | GGCAGAACGCTCCATTGTGTAT | — |
| GOT1 F | GTCCAGTACCACCAAAGTAGTTCTC | — |
| GOT1 R | GGCTCTAATCCCAGTCTCCAAA | — |
| ME1 F | TATACAGCTTGCGGAGGGATGAA | — |
| ME1 R | TGCCATACTTGGAAGAAACTGCC | — |
| MDH1 F | CTCAAGGGAGAATTTGTCACGACTG | — |
| MDH1 R | AGCAGATCATCAGGAACACCATAGG | — |
| GOT2 F | GGCTTATATGGTGAGCGTGTAGG | — |
| GOT2 R | TTTCGCAAATCTGGGGTGTTC | — |
| GLS1 F | CCTTCTGTCTTCAGTCCTGTGTAAA | — |
| GLS1 R | TTGAATCTTAGTCCACTCGGCTCT | — |
| GLS2 F | TCTTCACCCAGGTCTCCTCTTCC | — |
| GLS2 R | TAGCCATTCCCACTTCCCATATTC | — |
| β-actin F | GTTGCGTTACACCCTTTCTTGAC | — |
| β-actin R | CTCGGCCACATTGTGAACTTTG | — |
| si-HIF-2α#1 F | CUCUGGAUUUCGGGAAUCATT | siRNA |
| si-HIF-2α#1 R | UGAUUCCCGAAAUCCAGAGTT | — |
| si-HIF-2α#2 F | GGAGCUAACAGGACAUAGUTT | — |
| si-HIF-2α#2 R | CUAUGUCCUGUUAGCUCCTT | — |
| sh-HIF-2α#1 F | CTCTGGATTTCGGGAATC | shRNA |
| sh-HIF-2α#1 R | TGATTCCCGAAATCCAGAG | — |
| sh-HIF-2α#2 F | GGAGCTAACAGGACATAGT | — |
| sh-HIF-2α#2 R | CTATGTCCTGTTAGCTCC | — |
| F: Forward primer; R: Reverse primer | | |
